# Scalable method for bio-based solid foams that mimic wood

**DOI:** 10.1038/s41598-021-03764-0

**Published:** 2021-12-21

**Authors:** Mikael Reichler, Samuel Rabensteiner, Ludwig Törnblom, Sebastian Coffeng, Leevi Viitanen, Luisa Jannuzzi, Tero Mäkinen, Jonatan R. Mac Intyre, Juha Koivisto, Antti Puisto, Mikko J. Alava

**Affiliations:** Department of Applied Physics, Aalto University, P.O. Box 11100, 00076 Aalto, Espoo, Finland

**Keywords:** Mechanical properties, Statistical physics, thermodynamics and nonlinear dynamics

## Abstract

Mimicking natural structures allows the exploitation of proven design concepts for advanced material solutions. Here, our inspiration comes from the anisotropic closed cell structure of wood. The bubbles in our fiber reinforced foam are elongated using temperature dependent viscosity of methylcellulose and constricted drying. The oriented structures lead to high yield stress in the primary direction; 64 times larger than compared to the cross direction. The closed cells of the foam also result in excellent thermal insulation. The proposed novel foam manufacturing process is trivial to up-scale from the laboratory trial scale towards production volumes on industrial scales.

## Introduction

Nature can be used as an inspiration for finding ways to manufacture advanced materials with novel functionalities^1–3^. Wood is an example of such a material to copy: it is lightweight, strong, and can be used as raw material for many purposes that require mechanical or insulating properties. These features originate from the structure of wood, specifically its closed anisotropic cellular geometry^4^.

The fact that practically all cellular structures posses anisotropic mechanical properties has been known for decades^5^. The idea to mimic highly anisotropic wood structure to intentionally introduce extreme anisotropy to foams allowing one to control their mechanical properties has recently been successfully applied to, for instance bio^6^, carbon nanotube^7^, crosslinked HDPE, and natural rubber^8^ based foams. In the context of bio-based porous materials introducing anisotropy has been revoked as a method to improve their material properties^9–11^.

A wood-like, porous and oriented structure can be achieved multiple ways. The existing wood structure can be modified by chemical treatment of the cell walls creating a highly compressible carbon sponges^12^. Alternatively, if the original structure is destroyed, it can be reassembled to a larger scale by using 3D or 4D printing^13^. Also, sheets of graphene^14^ or cellulose (nano)fibers^15^ can be assembled into an oriented structure by growing ice crystals. Once the desired structure is obtained, the excess solvents and weight can be removed by freeze-drying and carbonating^16^.

In this work, we mimic wood by making dry foams from fibrous forest-based materials^17,18^. The essential part of the process is the hierarchical structure^19^, where a cellular structure with elongated bubbles^6^ couples to fiber orientation in the cell walls. We present an easily scalable novel process for the production of bio-based anisotropic foams and show that the solid foams thus obtained indeed possess mechanical properties exhibiting directional anisotropy and complex response to loading. The proposed process differs significantly from the freeze-casted or aerogel production by its ability to scale up even to a large paper machine level.

We start with a wet foam, which is then forced to deviate from the natural isotropic geometry. The transient property is transferred to the final dry foam by slowing down the cell shape relaxation down to the scale of our drying process, achieved by manipulating the fluid’s rheological properties. The novelty of our approach are the properties inherited from the manufacturing process, which allows cheap and industrially scalable biomimetic creation of lightweight materials from raw materials that appear to be a promising recyclable^20^ candidate for the substitution of plastics^21^ in many future applications^22–27^.

**Figure 1:**
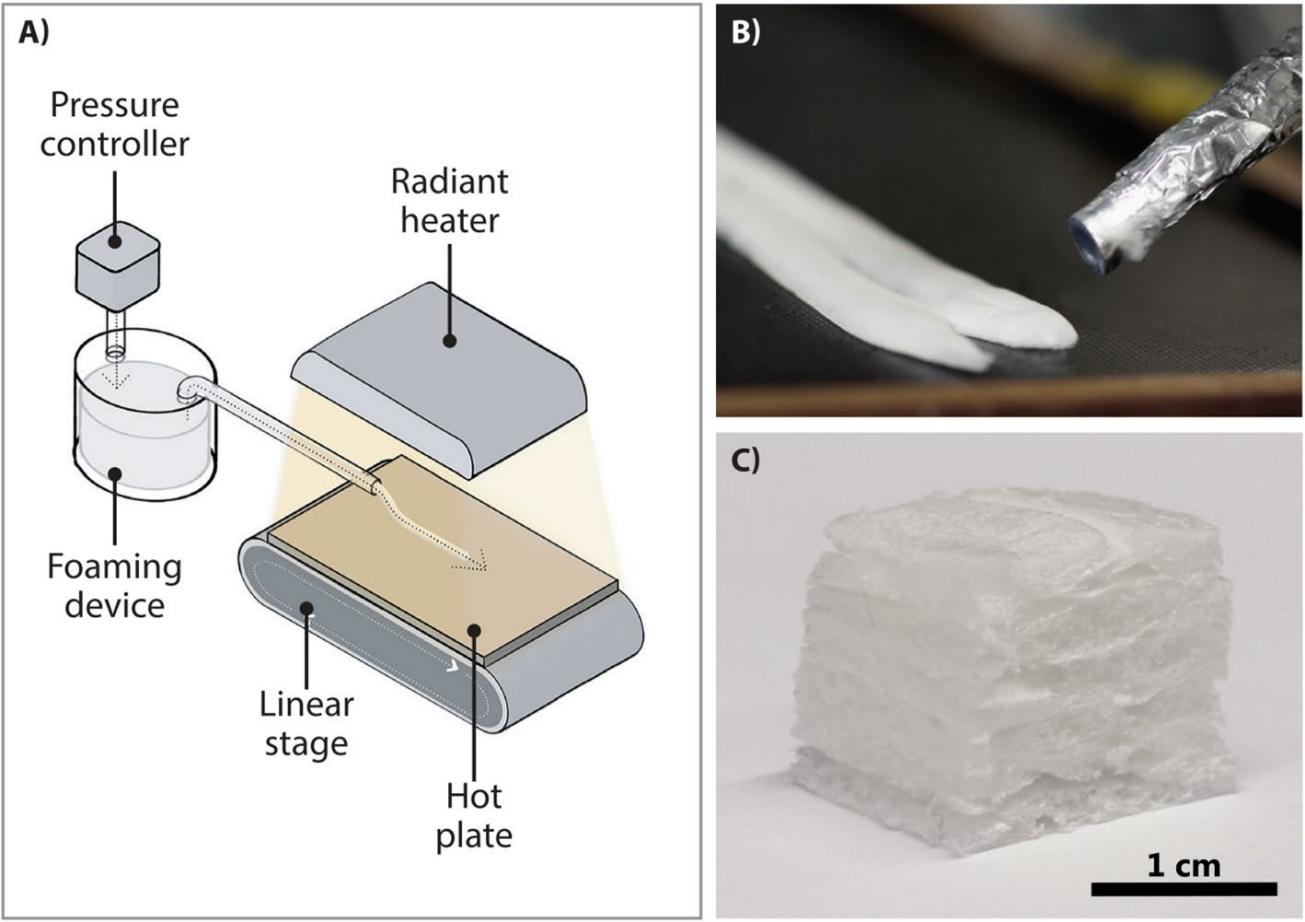
Making wood-like foams. (**A**) Schematic view of the setup showing how the foam is ejected from the foaming device by the controlled air pressure to the hot plate. The linear stage moves as the foam is ejected, creating a solid rod like object that is dried by the radiant heater. (**B**) Close view of two rods created by two passes of the cylindrical extruder head. (**C**) Final foam block cut into a cube for compression testing.

**Figure 2:**
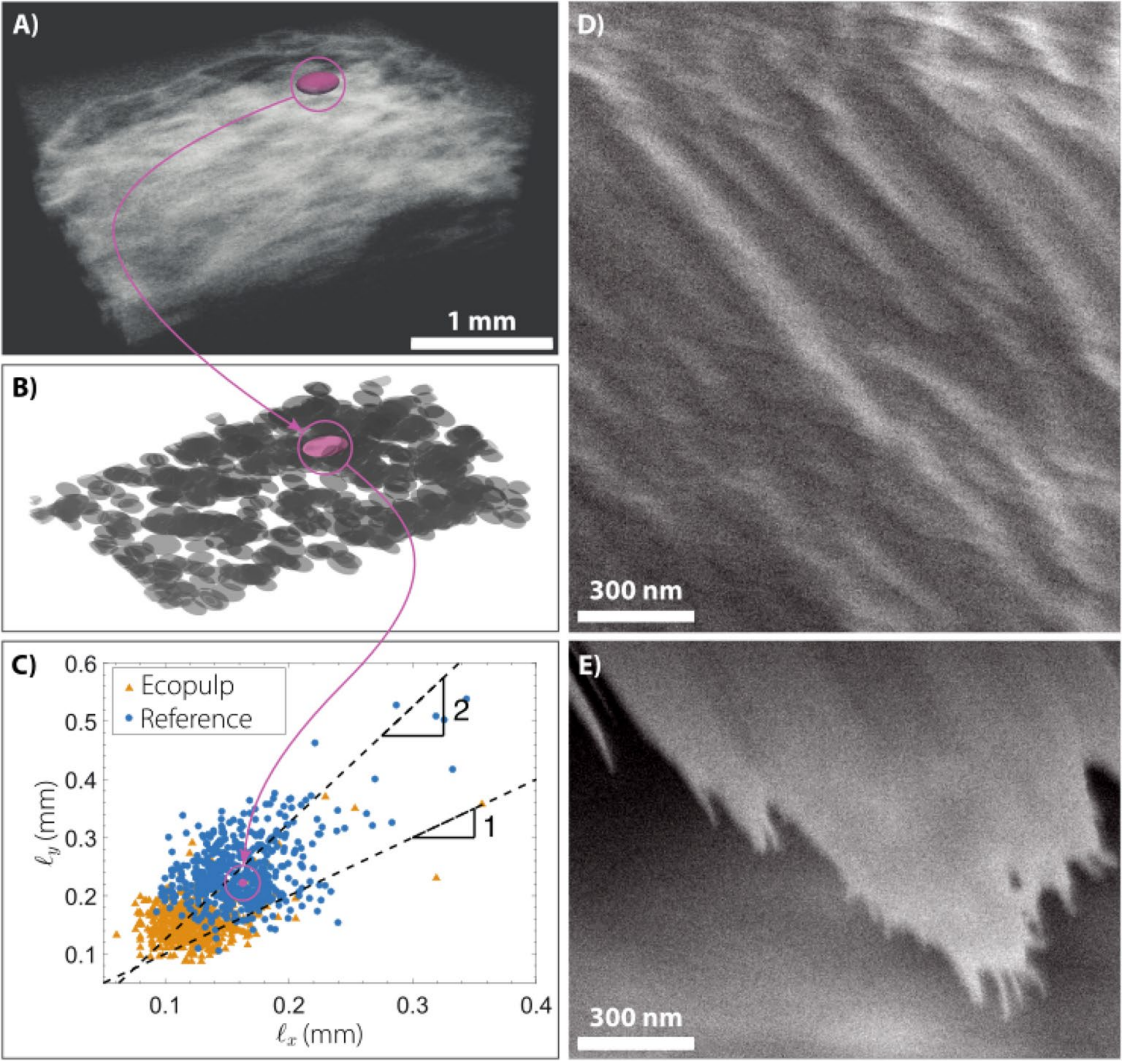
Structure of wood-mimicked foams. Microscopy image of a foam rod. (**A**) Reconstruction of the OCT signal provides a 3-dimensional internal structure of a single rod. The solid structure is shown in white making it appropriately transparent for clarity. (**B**) Visualization of the larger pores inside the rod represented with ellipsoids (negative space of **A**). (**C**) Pore dimensions $\ell _x$ vs. $\ell _y$. Black dashed line represents $\ell _x = \ell _y$. The correlation $\ell _y > \ell _x$ implies that the pores are elongated along the rod axis. (**D**) The fiber structure was explored by using Scanning Electron Microscopy. A high magnification over the solid structure of the foam block shows an oriented ridged distribution. (**E**) The nearby edge of the torn film has whiskers (white) oriented along the structures seen in Panel (**D**).

## Results

In general, the advantage of cellular materials is their combination of low density and excellent mechanical properties. The foam material we create follows this paradigm, which demonstrates particular differences depending on loading directions, or “soft” and “hard” orientations. We exploit forest-based (nano)materials: ordinary reference pulp fibers, EcoPulp MicroFibrillatedCellulose (EcoPulp) and TEMPO oxidized NanoFibrillatedCellulose (TEMPO)^28^ as the main structural components in our experiments. The structure and mechanical properties can be tuned to a large degree depending on the starting material and the process details.

The setup used to extrude rod-like foam structures is shown in Fig. 1A. The key elements are the continuous deposition process of rods from extruder head (shown in Fig. 1B) and the subsequent drying by a moving, heated surface and a radiant heater. Larger structures are achieved by subsequent deposition of further foam “rods” side-by-side and stacked layers that are then cut into blocks seen in Fig. 1C. In the drying process, bubble shrinkage is geometrically hindered in the axial, but not in the radial, direction: The rod shape and the friction between the rod and the surface reduce bubble shrinkage in the axial direction promoting radial shrinkage and thus fiber orientation.

### Microscopical properties

We examine the foam block structure using Optical Coherence Tomography (OCT) and Scanning Electron Microscopy (SEM). Figure 2A shows the 3-dimensional tomography image of the dry foam block, with the solid structure in white while the pores appear as empty space. Pores are mostly flat and elongated in the foam block plane (Fig. 2B). By reconstructing the OCT signal, we are able to detect each hole in the foam block surrounded by the solid structure. Then we effectively determine the dimension of each pore as the second central moment of the voxel group $\ell _i^2$, for $i = (x,y,z)$ (Fig. 2C). In this plot, most of the points are located above the symmetry axis $\ell _x = \ell _y$ associated with spherical pores (black dashed line). Therefore, on average the pores are elongated along the main axis of the extruded structure (the y-axis) with an aspect ratio around 1:2.

A high magnification SEM image (Fig. 2D), created by manually breaking a macroscopic TEMPO foam block and coating the fracture surface with 4 nm conductive Au-Pd nanoparticles, shows the TEMPO fibers on the dry solid structure. Visual inspection shows an oriented ridged structure, which we propose is a set of clustered nanofibers under the conductive coating. Figure 2E shows the edge of the same region where the ridges continue as “whiskers”, further confirming the oriented structures not only in the pores, but also on the (nano)fiber level. Similar orientation of CNF’s and CNC’s has been associated to excellent mechanical properties in 3D printed cellulose composite materials^29^.

### Compression experiments

To measure its deformation properties the sample is compressed between a compression piston and an acoustic emission (AE) sensor, ensuring a good acoustic coupling. This allows us to study the stress-strain curves in compression and to follow the microscopic deformation of the solid foam as well as determine yield stress as the strength of material. The AE data is transformed to a discrete set of events by setting a threshold for the amplitude *A*, and these “foamquakes” are characterized by the event energy.

**Figure 3:**
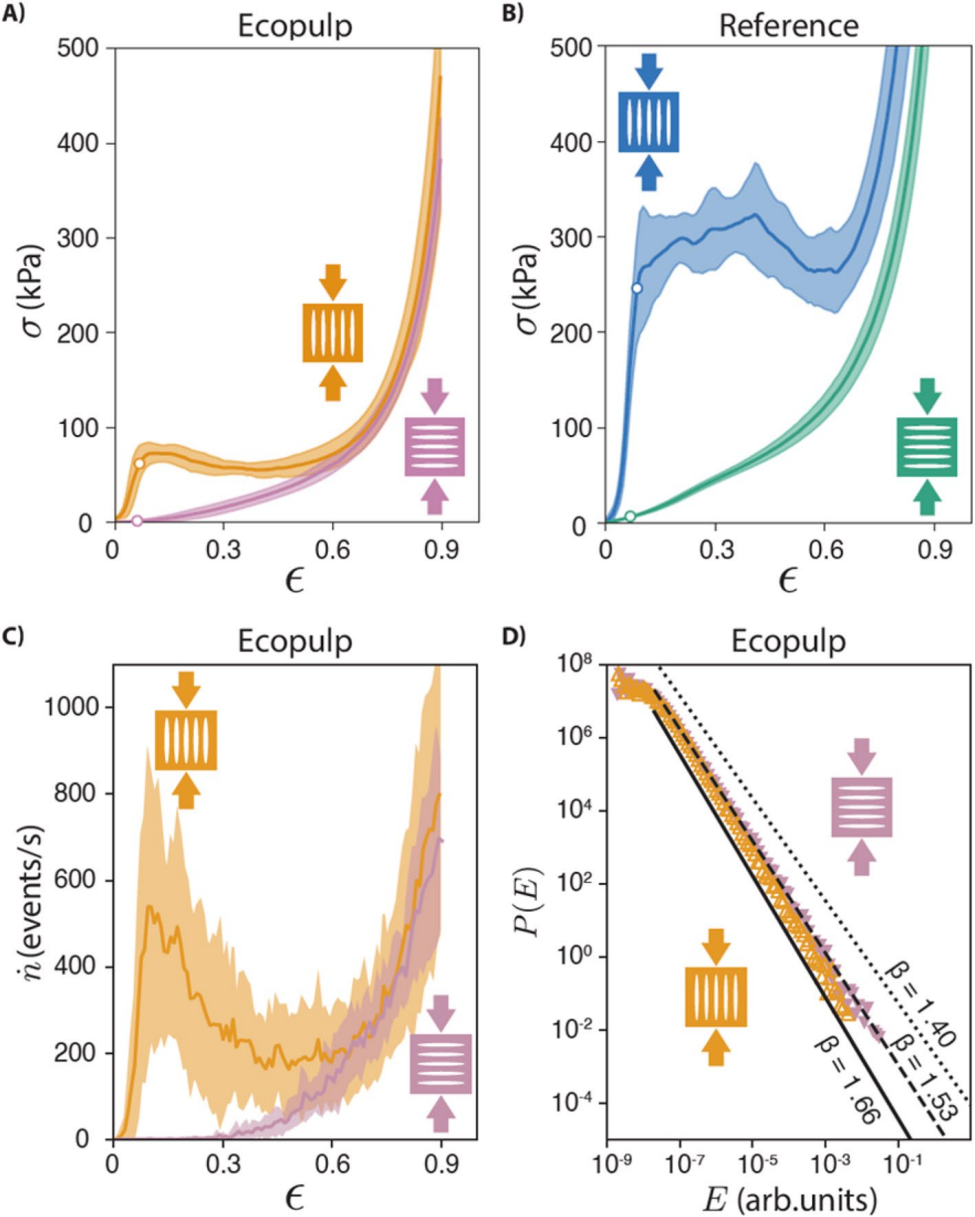
The mechanical properties of “Foamwood” and the complexity of deformation measured in acoustic emission by “foamquakes”. The compression data from Ecopulp fibers along the rod (yellow) and across the rod (pink). Reference fibers are shown along the rod (blue) and across the rod (green). Lines indicate the average of multiple experiments; the surrounding shaded area indicates the standard deviation. The yield point is indicated as circles in panels A and B. The anisotropy of the foam is particularly evident in the compression strength along and parallel to the major axis of the pores in both sample types, made from (**A**) chopped Ecopulp and (**B**) untreated reference fibers. (**C**) The acoustic emission (AE) event rate ${\dot{n}}$ indicates that the majority of pores oriented along the grain break near the yield strain at $\epsilon =10\%$, while the cross grain sample does not produce AE until $\epsilon =30\%$, indicating pure elastic deformation. (**D**) The theoretical AE energy probability distribution functions have three three models (black). There is a clear difference between experiments with different pore orientation.

The main results are shown in Fig. 3, which compares the properties of solid foams made of Ecopulp and MC (Fig. 3A) with those of reference fibers and MC (Fig. 3B). The compression strength of $\sigma _ y = 64$ kPa along the major axis of the pores for Ecopulp (yellow circle in Fig. 3A) is 64 times the strength in the cross direction (pink circle in Fig. 3A, $\sigma _y = 1$ kPa). The strength difference between the orientations is significantly larger by a factor of 10 compared to anisotropic cellulose aerogels^30^. Moreover, the long reference fibers have strength $\sigma _y = 248$ kPa (blue circle in Fig. 3B) which is 3 times the compression strength of the short Ecopulp fibers (yellow circle in Fig. 3A). In the soft direction, the compression strength of long fiber foam is $\sigma _y = 9$ kPa (green circle in Fig. 3B). The maximum specific strength (or strength per weight; $\sigma _y=248~\mathrm{kPa}/41~\mathrm{kg}/\mathrm{m}^3$), is obtained with long reference fibers and pores oriented along the compression (blue). The elastic moduli can only be determined along the major axis and those are $Y=1.4$ MPa and $Y = 5.5$ MPa for Ecopulp and reference. The values of *Y* and $\sigma _y$ on hard direction are close in magnitude to previous polysaccharide based foam materials with similar densities^31^.

The acoustic emission is measured to probe the details of sample irreversible deformation in terms of the number and energy of the intermittently emitted acoustic wave bursts (events) during the compression. Here it is specifically used to show the differences under uni-axial compression on directions parallel and perpendicular to the anisotropy (and pore) orientation. Figure 3C shows the AE event rate, which was observed to be much larger with the reference fibers and when the compression is along “the grain” or the elongation axis of the pores (blue). There are thus many more contacts breaking^32^ before the material yields (at yielding, the AE rate peaks). Looking at the total number of events in the compression in different directions (see Methods for details) shows that in the hard direction the number of events is roughly double compared to the soft direction.

The AE energies, or crackling noise avalanche sizes^33^, are distributed as a power-law $P(E) \propto E^{-\beta }$. This type of behavior under compression has been observed for many different materials/systems ranging from earthquakes^34^ and rock fractures^35^ to porous silica glass (Vycor)^36^, wood that we mimic^37^ and charcoal^38^. We find that the distribution of acoustic emission energies is power-law distributed spanning five to six decades in energy (Fig. 3D). The exponent $\beta$ differs between compression directions. In the harder direction (along the rod), the exponent aligns with values expected for more heterogeneous materials ($\beta = 1.66 \pm 0.01$), while in the softer direction (across the rod) the exponent falls between the two suggested classes ($\beta = 1.53 \pm 0.01)$, suggesting a combination of homogeneous and heterogeneous material behavior^38^. This indicates that the failure mode of the samples compressed in different directions is different, reflecting the difference of the material structure depending on the compression direction.

This classification has been formulated for compression of brittle materials, so the usefulness in this case is remarkable. A parallel for this relationship between wildly varying materials is the exponent of the “homogeneous material” class $\beta = 1.4$ which has been observed i.e. for brittle Vycor^36^ and viscoelastic wood^37^. Here homogeneity refers to the small pore size in the samples.

### Thermal conduction

To measure the thermal conduction, we use a thermal imaging method previously applied to wood samples^39^. Since it is based on an infrared (IR) camera, it also allows potential homogeneities in the samples to be detected. A cubical block of the sample is added on top of the copper heat plate (which reflects IR radiation) with a constant temperature $T = 69^{\circ }$C and imaged with a thermal camera, resulting in a temperature profile with heat flux $\alpha =5 \cdot 10^{-7}~{\mathrm{m}^2/\mathrm{s}}$. The heat flux *T*(*y*) solution is fitted to the profile data, and the thermal conductivity *k* is then obtained from $k = \alpha \rho C = 0.029~\mathrm{W}/(\mathrm{m}\, \mathrm{K})$, where $\rho =41 ~\mathrm{kg}/\mathrm{m}^3$ is the density and $C=1400~\mathrm{J}/(\mathrm{kg}\, \mathrm{K})$ is the specific heat capacity of typical cellulose. The obtained result is verified using the modified transient plane method. This yielded slightly higher thermal conductivities of $k = 0.035$ to $0.037 ~{\mathrm{W}/(\mathrm{m} \, \mathrm{K})}$ on the perpendicular direction to the pore major axis and $k = 0.039 ~{\mathrm{W}/(\mathrm{m} \, \mathrm{K})}$ on the parallel direction to the pore axis. This reveals that the material is better at insulating than felt (thermal conductivity $k = 0.07~{\mathrm{W}/(\mathrm{m}\, \mathrm{K})}$) or wood such as the low-density balsa, which has a thermal conductivity ranging from $k=0.034$ to $0.067~{\mathrm{W}/(\mathrm{m}\, \mathrm{K})}$^40^.

## Discussion

**Figure 4:**
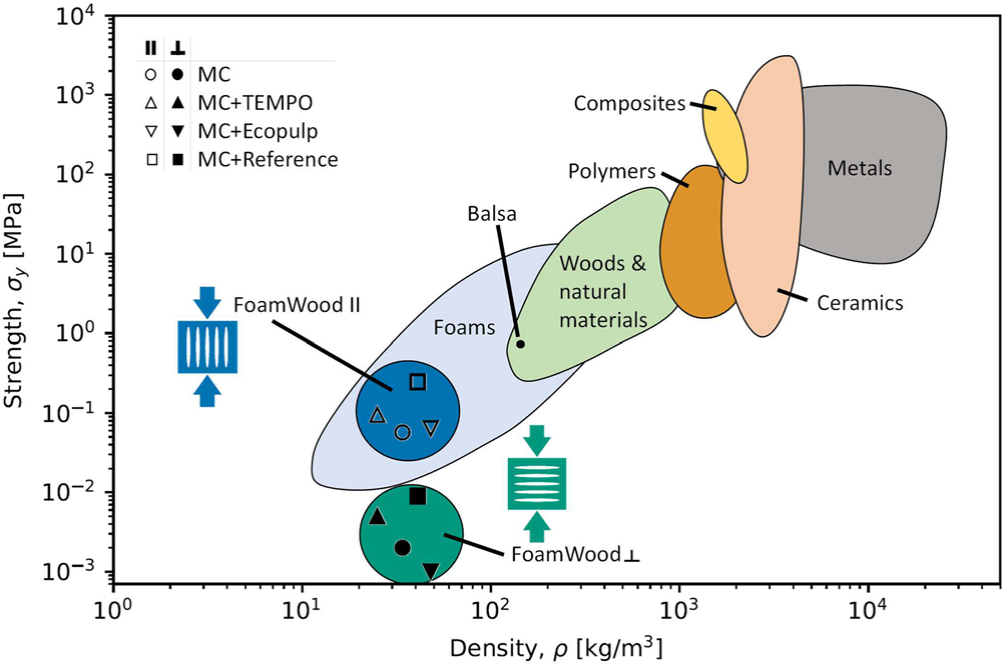
Material properties of wood-like solid foams: a comparison of the density vs. strength. An Ashby-type plot illustrating the relation of strength vs. density of FoamWood as well as other engineering materials. The colored areas are reproduced from Ref.^41^.

Creating plastics alternatives out of renewable materials remains a challenge for two reasons. Firstly, the ease of manufacturing and variety of oil-based materials make them attractive, and secondly, they display refined material properties. We have presented here a biomimicry based idea of how to create foams that have wood-like properties: elongated closed pore cellular structures with oriented particles inside the pore walls, and consequently anisotropic mechanical behavior.

The material produced by our process is both, soft and very hard depending on its loading direction as seen in the “Ashby”-plot (Fig. 4) showing how our experimental data of these foams compare with other solids, foams and wood in terms of strength versus density. Such a comparison to other materials highlights the promise of using forest-derived raw materials and biomimicry to pave the way to competitive alternatives for fossil-oil based products.

The proposed simple and scalable manufacturing process creates a hierarchical structure where the macroscale orientation of foam when extracted from the wet state is inherited into the mesoscale pores and again in the microscale fibers. Adding functionality, such as folding structures or stress redistribution would be possible either by laminating layers with different orientation or extruding shapes other than rods, e.g. Z, H or V-shapes.

The bio-based fibers in the bubble film could also be replaced by or composited with other materials. Some of the most interesting alternatives would be plate like particles, such as clay or graphene. Then, the assumption would be that the strength of the material should significantly increase without compromising the lightweight structure.

In summary, we have created a wood-like foam, which has application potential as a plastics substitute. This recipe has as it is physical properties of interest and allows for tuning in order to optimize e.g. the strength to weight ratio or to make it exploitable for specific purposes.

## Methods

**Figure 5:**
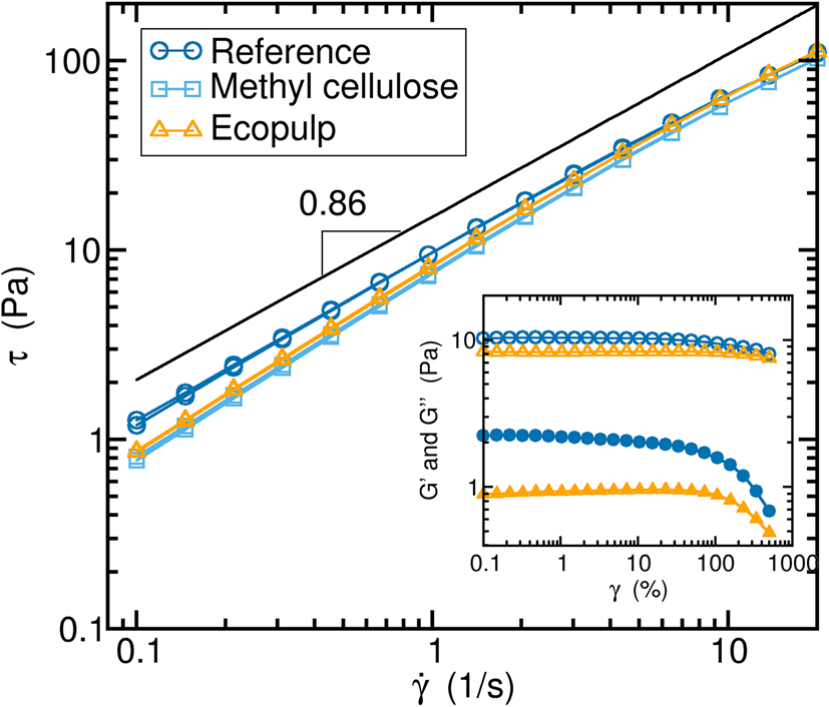
Rheological properties of the suspensions. Flow curve of a pure MC solution, with squares showing typical shear thinning behavior, where the shear stress $\tau$ is plotted against the strain rate ${\dot{\gamma }}$. Adding the short Ecopulp fibers (triangles) increases the viscosity by a constant, but does not affect the shear thinning behavior. On the other hand, the long reference fibers (circles) slightly increase the shear thinning, as seen in change of slope of the curve and hence in the shear thinning exponent. The inset shows the storage $G'$ (filled symbols) and loss $G''$ (unfilled symbols) modulus of the reference and Ecopulp fibers over the shear amplitude $\gamma$.

Low solid content foams were prepared from three types of mixtures: untreated reference pulp, microfibrillated cellulose (Ecopulp) and nanofibrillated cellulose (TEMPO) fibers^42^, each one in combination with ordinary food grade MethylCellulose (MC). The latter works as a gelling agent if heated to $T_g =70^{\circ }$C, and is used to tune the rheology of the foam extrusion and increase the relaxation time associated with the recovery of the isotropic structure. The high viscosity found for the shear rate of the extrusion speeds ($\sim$0.1 1/s) is ideal, as it prevents the elongated bubbles created in the extrusion from changing shape.

### Properties of the bulk suspension

The dry weight of fibers—both untreated reference fibers and enzymatically chopped Ecopulp fibers—is significantly low $w_{fib}=0.16$ mass% compared to the $w_{mc}=2.5$ mass% of MC or $w_{H_2O}=97.34$ mass%. These concentrations result in densities of 48 and 41 kg/m$^3$ for final Ecopulp and reference fiber foams. The low concentrations needed are explained by the chemical properties of the suspension at high temperatures. For example, the food grade MC used here forms a gel (polymerizes) at $T_g =70^{\circ }$C due to its modest degree of substitution ($d_{sub}=1.7\pm {0.1}$). There are other grades of MC with lower gelifying temperatures (e.g. $37^{\circ }$C) and higher degrees of substitution. The degree of substitution defines the amount of hydroxyl groups (-OH) replaced by methoxide groups (-$\hbox {OCH}_3$) in the methylcellulose molecule. In practise, these replaced hydroxyl groups bind the cellulose molecules together at high temperatures producing a strong gel-like network. In this application, the MC tunes the rheology during the foam extrusion and significantly increases the relaxation time of the foam, allowing the fibers to orient and bind or entangle together.

In addition to the two fibrous foams, one foam is prepared without any added fibers using only methyl cellulose. The concentration methyl cellulose remains the same $w_{mc}=2.5$ mass% and the resulting density of dry foam is 35 kg/m$^3$. Also, foam prepared with TEMPO oxidized nanocellulose was experimented. The high viscosity of TEMPO suspension allowed only fiber concentration $w_{nfc}=0.06$ mass% and lower concentration of MC $w_{mc}=1.0$ mass%. The small cellulose and MC concentrations in the bulk decreased the density of the final foam samples to 25 kg/m$^3$.

The original source of all fibers is Norway Spruce, *Pinea Abies*. The Ecopulp fibers are chopped with a commercial Ecopulp enzyme for 240 minutes to significantly reduce the average fiber length. Standard Finnish food grade high purity, non-distilled tap water is used as the background fluid.

The rheological properties of the bulk (not foamed) suspensions were determined using a concentric cup and bob geometry, on an Anton-Paar MCR302 rheometer. The results are shown in Fig. 5. High viscosity in the range of the shear rate of the extrusion speeds ($\sim$ 0.1 1/s) is preferred, as it prevents the elongated bubbles created in the extrusion from changing shape. This is best obtained with the reference fibers (dark blue circles).

In addition, the Ecopulp fibers (triangles) did not appear to affect the shear thinning of the MC suspension (squares). We conclude that the Ecopulp fiber length is too small to create networks and the methyl cellulose is dominating the solution. In contrast, the longer reference fibers dominate the suspension rheology for low shear rates. The same is seen in large amplitude oscillatory shear experiments in Fig. 5 inset; the storage modulus $G'$ deviates from Ecopulp fibers mostly with small strains, indicating that the fiber network is carrying load.

Here, we hypothesize that at higher viscosity or loss of modulus, more anisotropy is stored in the end product, as the fiber network is still intact. In addition, a very slow relaxation process of the elongated bubbles compared to the drying speed (measured in minutes) is preferred.

**Figure 6:**
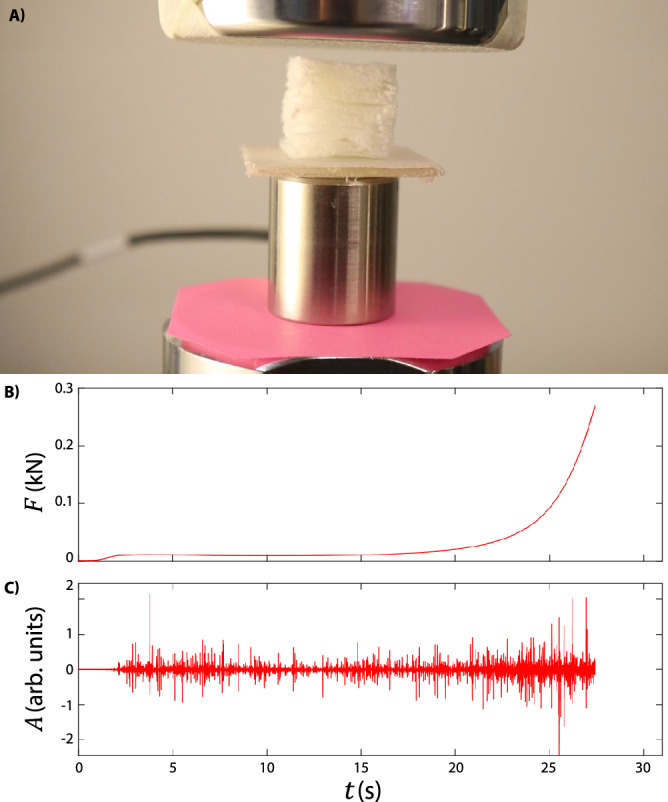
Compression protocol. (**A**) Sample between the two compression pistons of the Instron E1000 compression device. The pink paper at the bottom and the tape at the top break the acoustic connection between the machine and the AE sensor (steel cylinder). The sample is firmly attached to the AE sensor with double-sided tape to avoid sliding. The measured raw force (**B**) and AE amplitude (**C**) indicate a constant activity of structures breaking.

### Foam extrusion method

Figure 1 shows the setup used to create the rods. The foam is deposited onto a solid surface from a nozzle. The final product is ejected from the foaming device, which consists of a sealed cup and spinning blade, to the solid plate surface using a pressure controller (pressure $\Delta P = 0.1$ Pa). As the foam is deposited, the solid plate moves linearly, creating a rod of $l = 150$ mm in length and $w = 6$ mm in width. The hotplate moves at a speed of $v= 1$ mm/s. The process is repeated until 10 rods are deposited onto the solid plate, side by side. The rods are dried using a radiant heater. Once the rods are fully dried, another layer is created on top of the existing layer.

Here, the essential physics are described by the balance between foam flow rate, hotplate moving speed, radiant heater distance, as well as bubble and fiber relaxation times of the foam. The goal is to create a flow in the extrusion elongating the bubbles and gellify the MC by heat, allowing the fiber network to stabilize during drying. Furthermore, the rod shape prevents the bubbles from shrinking in the axial direction, preferring radial shrinkage instead. The hotplate moving speed is determined to be the maximum speed (with stability and usability marginal) before the bubbles in the foam start to switch neighbors and the rod breaks into two pieces.

### Compression measurements

The compression setup is depicted in Fig. 6A, which shows the sample compressed between a compression piston and an acoustic emission sensor to ensure a good acoustic coupling. To obtain uniform compression, a thin plastic plate, wider than the sample, was placed between the sample and the AE sensor. The AE sensor is a wideband sensor F30$\alpha$ connected to a preamplifier by Physical Acoustics corporation. We have verified that under these small loads, the steel and ceramic casing of the sensors is rigid within the measurement accuracy and is not a source of acoustic emission. The amplified signal is then digitized by a National Instruments 6040E digital acquisition card with a sample rate of $f = 100$ kHz. The compression piston is connected to an Instron Dynacell load cell, which is connected to the Instron E1000 tensile testing machine that digitizes the load-displacement data in compliance with ASTM standards. The raw force and AE amplitude signals are shown in panels B and C of Fig. 6. For the Ecopulp samples 5 experiments were conducted for both the soft and hard compression directions, and for the reference samples 3 in the soft direction and 4 in the hard direction.

The raw acoustic emission signal is transformed to a discrete set of events by setting the threshold of the amplitude *A* to just above the noise level. For each event, which is part of the signal above this threshold, the event energy is calculated (in arbitrary units) as

1 $$\begin{aligned} E = \int A^2 \, \mathrm{d} t, \end{aligned}$$

where the integration is done over the duration of the event. The Ecopulp samples produced $5721 \pm 1585$ events per experiment when compressed in the soft direction and $10526 \pm 4239$ events per experiment when compressed in the hard direction, yielding then datasets of tens of thousands of events for both directions. The threshold level used is at least two orders of magnitude below the peak amplitude of the highest acoustic emission peaks, thus enabling the detection of a wide range of acoustic event energies.

These energies are distributed as a power-law $P(E) \propto E^{-\beta }$, and the value of the exponent $\beta$ is estimated using the method of maximum-likelihood maps^36^. In this method, a maximum-likelihood estimation for the exponent is done between values $E_{low}$ and $E_{high}$. These two values are varied, producing a map of exponent values. By discarding areas where the error estimate of the maximum-likelihood estimation is too high (here 0.05), an error margin $\delta \beta$ can be set for the exponent (here 0.01) and then the exponent with the largest area of the map corresponding to $\beta \pm \delta \beta$ can be identified.

**Figure 7:**
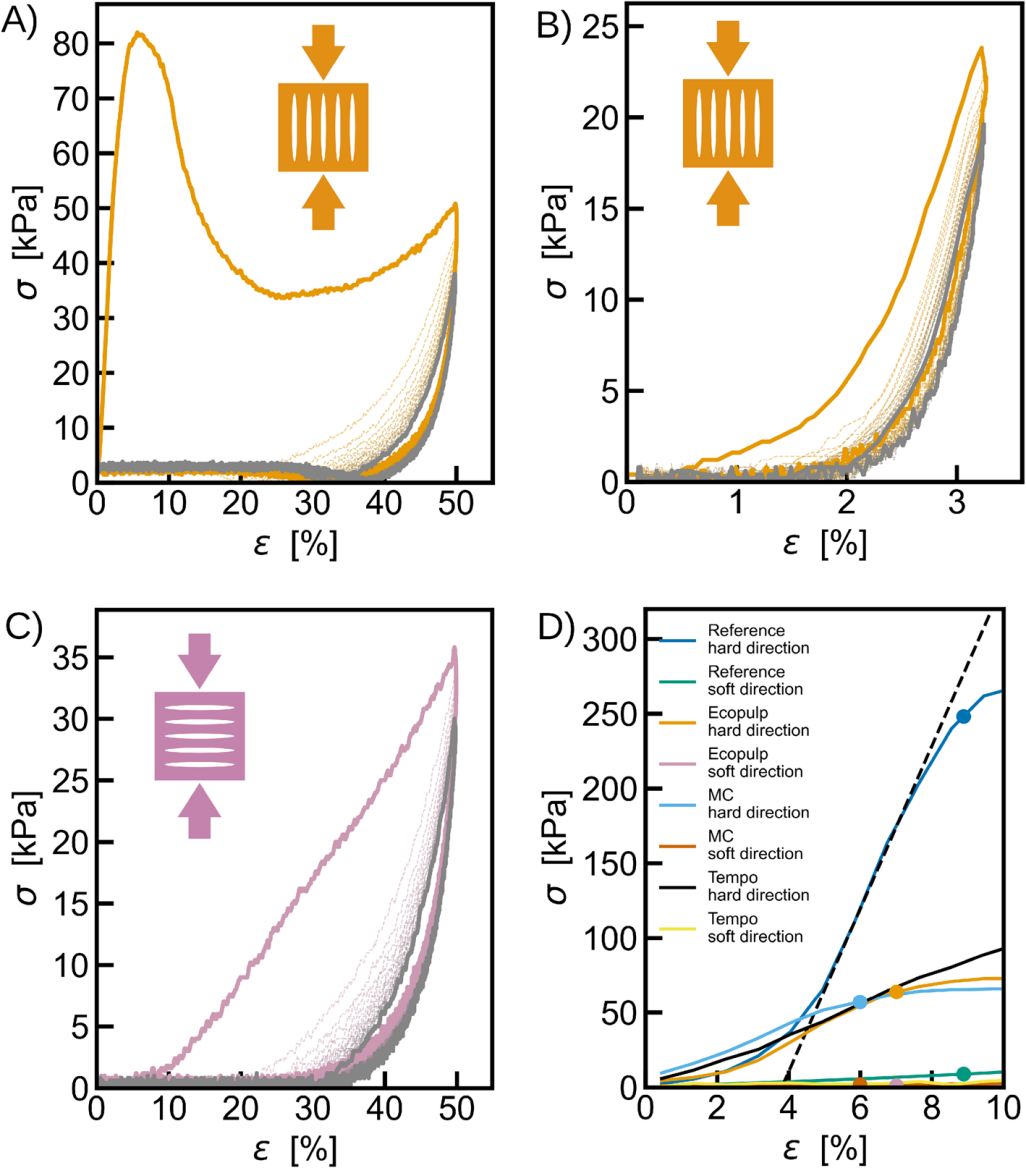
Determining the strength of material. (**A**)–(**C**) The elastic response of the material was tested on cyclic compression experiments with 20 cycles. The coloured solid lines represent the first cycle and the gray solid lines the 20th cycle. The samples were produced from Ecopulp and they were tested on the hard direction with (**A**) 50% compression and (**B**) 3.5% compression. (**C**) Similar test was conducted for soft direction with 50% strain. (**D**) The beginning part of the average stress-strain curves showing the determination of the yield stress (dots) using the linear fit to the maximum slope of the elastic part (black dashed line as an example). In the soft direction the yield stress is determined as the stress value corresponding to the strain which equals the yield strain of the compression in the hard direction.

The applied stress $\sigma$ is derived from the load-displacement data by dividing the load by the initial cross-sectional area of the sample perpendicular to the compression direction. Similarly the strain $\epsilon$ imposed on the sample is derived by dividing the displacement by the initial height of the sample. First, the elasticity of material is explored in cyclic compression tests with 20 cycles for the Ecopulp samples. The results for the hard direction are shown in Fig. 7A and B for maximum strains of 50% and 3.5%, respectively. These indicate the destruction of the structure if the sample is compressed beyond yielding. However, with modest strain value the structure remains mostly intact showing elastic response. The soft direction lacks elastic response as seen in Fig. 7C and the stress response is only detected after the structure starts to yield.

The yield stress (for compression in the hard direction) is then calculated by fitting a straight line to the maximum slope of the elastic part of the stress-strain curve (see Fig. 7D) and defining the yield stress to be the point where the stress deviates 10% from this line. The slope of this line corresponds to the elastic modulus *Y* of the material. As no clear elastic part of the stress-strain curve can be seen in the compression in the soft direction, the yield stress in this case is defined as the stress corresponding to the yield strain in the hard direction (the strain corresponding to the yield stress).

For sanity check, similar compression experiments than in Fig. 3 were performed with foam made of pure MC suspension with no fibres and foam with MC and TEMPO oxidized cellulose nanofibers (Fig. 7D). Compared to Ecopulp the pure MC foam had slightly smaller compression strength of $\sigma _y = 57$ kPa and elastic modulus $Y=1.1$ MPa along the major axis of pores. In perpendicular to the major axes the compression strength dropped to $\sigma _y = 2$ kPa. The comparison to fibrous material shows that the pore structure and the orientation of fibers enhance the compression strength and elastic modulus along the major axis and the fiber direction. While on the direction perpendicular to the fibers the effect of fibers is smaller. With the CNF foam, however, the strength on the soft direction was improved more than on the hard direction: the compression strengths were $\sigma _y = 5$ kPa and $\sigma _y= 95$ kPa respectively.

### Determining the thermal conductivity

**Figure 8:**
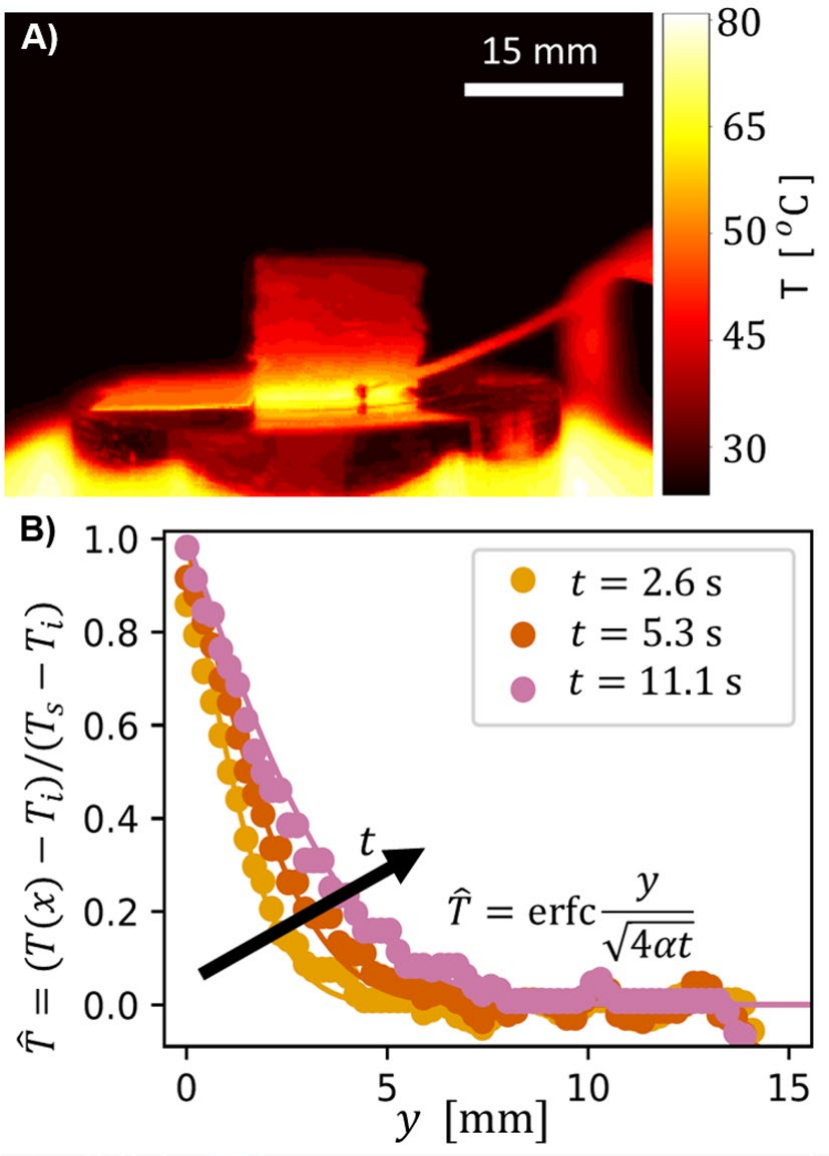
Heat conductivity measurement. (**A**) Unprocessed image from the IR camera shows the heat propagation through the cubic sample at the center. (**B**) Temperature profile from the center of the sample obeys typical form of $(T(y) - T_i)/(T_s -T_i) = \mathrm{erfc}(y/\sqrt{4\alpha t})$ where $\alpha = 5 \cdot 10^{-7}~\mathrm{m}^2/\mathrm{s}$ is the heat flux. The heat front profile propagates from the orange curve to pink curve in 7 s, allowing the estimation of the heat flux.

To measure the thermal conduction properties, we use the same technique utilized in Ref.^39^ where a cube of reference fiber foam is added on top of the heat plate at a constant temperature $T = 69^{\circ }$C and imaged with a thermal camera. A raw thermal image is shown in Fig 8A. The sample highlighted in the dashed square is on top of a hot copper plate (which reflects IR radiation). The temperature of the plate is measured with a thermocouple, shown as a rod and ball in front of the sample. The temperature profile shown in Fig. 8B is taken vertically from the center of the sample.

A heat flux *T*(*y*) in a 3D-object in time obeys an equation with a complement of the error function^39^

2 $$\begin{aligned} \frac{T(y) - T_i}{T_s -T_i} = \mathrm{erfc}\Big (\frac{y}{2\sqrt{\alpha t}} \Big ), \end{aligned}$$

where the $T_i$ is the initial ambient temperature, $T_s$ is the temperature of the surface, and $\alpha = 5 \cdot 10^{-7}$ m${}^2$/s is the coefficient of the heat flux.

In addition, the thermal conductivity results are verified with C-Therm TCi thermal conductivity analyzer. The device uses modified transient plane source method to determine the thermal conductivity. The method has been applied previously on analyzing the thermal conduction of insulators with comparison to other methods in Ref.^43^.
